# Henipavirus in Northern Short-Tailed Shrew, Alabama, USA

**DOI:** 10.3201/eid3108.250401

**Published:** 2025-08

**Authors:** Viola C. Haring, Sandra Diederich, Martin Beer, Florian Pfaff

**Affiliations:** Friedrich-Loeffler-Institut, Greifswald–Isle of Riems, Germany

**Keywords:** Henipavirus, viruses, Northern Short-Tailed Shrew, Alabama, USA

**To the Editor:** The article “Henipavirus in northern short-tailed shrew, Alabama, USA,” (*1*), describing the discovery of Camp Hill virus (family *Paramyxoviridae*) in the northern short-tailed shrew (*Blarina brevicauda*), sparked major media attention and raised concerns about zoonotic transmission and potential pandemic risk. However, it would be advisable to reevaluate this virus discovery within the broader context of related viruses. The increase in identified henipa-like viruses in various shrew species (*2*–*4*) led the International Committee on Taxonomy of Viruses to classify these henipa-like viruses into a distinct genus, *Parahenipavirus* (*5*), acknowledging their genetic difference from the highly pathogenic Hendra and Nipah virus.

Parahenipaviruses appear to be abundant in white- and red-toothed shrew species globally, but reports of infections in nonshrew species are limited so far, raising questions of their potential for spillover. Of note, no human infections with the Camp Hill virus have been reported to date, which aligns with the authors’ statement. The only known related shrew virus, which was detected in febrile, hospitalized humans and later in Ussuri and Shantung white-toothed shrews, was Langya virus (LayV) in China (*4*). The relationship between Camp Hill virus and its supposed reservoir suggests a great evolutionary distance between LayV and the crocidurine shrews. Of note, Hasua virus, a virus discovered in a white-toothed shrew in Germany (*3*), is genetically much closer related to LayV, but there is currently no evidence of associated human infections.

Because of the limited understanding of parahenipaviruses and the lack of evidence for their zoonotic potential, we urge caution in assuming pandemic risks. The absence of viral isolates and serologic studies are major limitations, underscoring the need for future research to guide risk analysis and response strategies.

